# Role of cardiac MRI in predicting the risk of right heart failure in patients who underwent left ventricular assist device implantation

**DOI:** 10.1016/j.jhlto.2024.100056

**Published:** 2024-01-15

**Authors:** Abdulelah Nuqali, Tarun Dalia, Grant McKinley, Taher Tayeb, Jinxiang Hu, Xiaohang Mei, Thomas Rosamond, Zubair Shah

**Affiliations:** a Queen's Heart Institute, The Queen's Medical Center, Honolulu, Hawaii; b Department of Cardiovascular Medicine, The University of Kansas Health System, Kansas City, Kansas; c Department of Internal Medicine, Division of Cardiology, Louisiana State University Health Shreveport, Shreveport, Louisiana; d Department of Biostatistics and Data Science, The University of Kansas Health System, Kansas City, Kansas

**Keywords:** cardiac magnetic resonance imaging, left ventricular assist device, right heart failure, right ventricular ejection fraction, mechanical circulatory support

## Abstract

Despite the advancement in the left ventricular assist devices (LVADs), right heart failure (RHF) remains a challenging adverse event after LVAD implantation and is associated with increased morbidity and mortality. In this study, we sought to assess the role of cardiac magnetic resonance-derived right ventricular ejection fraction (CMR-RVEF) in predicting the risk of post-LVAD RHF. Overall baseline characteristics and clinical outcomes were compared between the patients who developed post-LVAD RHF and those who did not. A total of 42 patients who underwent CMR before LVAD implantation were included in this study. The mean CMR-RVEF was 25 ± 13%, with no statistically significant difference between the 2 groups (27.7 ± 13.9% vs 24.5 ± 12.3, *p* = 0.5). The mean of the CMR-derived right ventricular volume index trend was higher in those with post-LVAD RHF (76 ± 28 ml/m^2^ vs 65 ± 25 ml/m^2^; *p* = 0.31). In conclusion, in patients who underwent CMR before LVAD implantation, CMR-RVEF may not predict post-LVAD RHF. Large multicenter studies are needed to confirm this finding.

Right heart failure (RHF) is a common adverse event after left ventricular assist device (LVAD) implantation and is associated with poor clinical outcomes.^1^ RHF post-LVAD implantation prevalence ranges between 9% and 42%, depending on the diagnostic criteria used to define RHF.^2^ Early identification of predictors for RHF is pivotal in the patient's selection for LVAD. Multiple clinical, hemodynamic, and echocardiographic parameters were studied to assess the risk of post-LVAD RHF, but it remains challenging. Cardiac magnetic resonance (CMR)-derived right ventricular ejection fraction (CMR-RVEF) is a promising surrogate of the right ventricular function.

In this study, we aim to assess the prognostic value of CMR-RVEF in predicting post-LVAD RHF. This retrospective, single-center study was conducted at the Kansas University Medical Center. We included all adult patients (≥18 years old) who underwent LVAD implantation and had CMR prior to LVAD implantation between January 2016 and December 2021. The criteria for post-LVAD RHF were adapted from the Interagency Registry for Mechanically Assisted Circulatory Support Mechanical Circulatory Support, Academic Research Consortium, which classifies RHF as early acute RHF, early postimplant RHF, and late RHF.^3^ CMR images were acquired using 1.5 and 3 Tesla clinical scanners. Standardized CMR imaging protocols followed the Society of Cardiovascular Magnetic Resonance guidelines.^4^ We used commercially available software to quantitatively analyze the chambers' volumes (cvi42; Circle Cardiovascular Imaging Inc., Calgary, Canada).

The study cohort consists of 42 patients who had CMR prior to LVAD implementation. Baseline characteristics are summarized in Table S1. The mean age was 47 ± 14 years, and 76% of the patients were males. Most patients were on milrinone infusion before LVAD implantation (69%). The most implanted LVADs in our cohort were HeartMate 3 (50%) and HeartWare (43%). About half of the patients were on the bridge-to-transplant strategy and half on the destination therapy strategy. CMR was obtained on an average of about 7 months prior to the LVAD implant. The mean CMR-RVEF was 25 ± 13%, with no significant difference between the 2 groups (27.7 ± 13.9% vs 24.5 ± 12.3, *p* = 0.5). The right ventricular end-diastolic volume index in the patients who had post-LVAD RHF was 105 ± 31.9 ml/m^2^, compared to 86 ± 31.1 ml/m^2^ in the patients who did not have post-LVAD RHF (*p* = 0.133). Table 1 summarizes the baseline CMR findings. The mean echocardiography-derived left ventricular ejection fraction in the patients who had post-LVAD RHF was 13.9 ± 6.5%, compared to 17.5 ± 7.2% in the patients who did not have post-LVAD RHF (*p* = 0.098). Severe right ventricle (RV) systolic dysfunction was more prevalent among the patients who had post-LVAD RHF (11.1% vs 6.1%; *p* = 0.05). The mean tricuspid annular plane systolic excursion was slightly reduced at 16 ± 4 mm with no significant difference between the 2cohorts (Table S2). The mean arterial pressure at the time of the RHC was lower in the post-LVAD RHF cohort at 76 ± 10 mm Hg, compared to 85 ± 12 mm Hg (*p* = 0.03). The cardiac indices were low by both Fick's calculation 1.85 ± 0.5 and thermodilution 1.54 ± 0.08, and the right ventricular stroke work index was low at 5.2 ± 2.8 gm/beat/m^2^. The pulmonary artery pulsatility index was 3.82 ± 4.35 in the post-LVAD RHF, compared to 3.28 ± 3.91 in the group who did not (*p* = 0.43) (Table S3). Nine patients developed post-LVAD RHF (21%); none had early acute RHF, 4 had early postimplant RHF, and 5 had late RHF. The overall mortality rate was 19%, with no significant difference between the 2 groups. The mean follow-up from the time of the CMR to the last clinical encounter was 31 ± 25 months.

**Table 1:** Baseline Cardiac Magnetic Resonance Characteristics

Cardiac magnetic resonance parameters	No right heart failure (N = 33)	Right heart failure (N = 9)	Overall (N = 42)	*p*-value
Time from CMR to LVAD (months)				
Mean (SD)	5.09 (13.0)	13.4 (25.4)	6.88 (16.5)	0.937
RVEF				
Mean (SD)	24.5 (12.3)	27.7 (13.9)	25.2 (12.5)	0.5
Right ventricular end-diastolic volume index (ml/m^2^)				
Mean (SD)	86.0 (31.1)	105 (31.9)	90.0 (31.9)	0.133
Right ventricular end-systolic volume index (ml/m^2^)				
Mean (SD)	65.3 (25.1)	75.8 (28.4)	67.6 (25.9)	0.312
TAPSE (mm)				
Mean (SD)	15.2 (5.14)	16.6 (4.56)	15.5 (4.99)	0.46
LVEF				
Mean (SD)	16.4 (5.79)	18.3 (11.7)	16.8 (7.33)	0.713
Left ventricular end-diastolic volume index (ml/m^2^)				
Mean (SD)	164 (57.1)	164 (39.9)	164 (53.4)	0.771
Left ventricular end-systolic volume index (ml/m^2^)				
Mean (SD)	130 (35.0)	136 (44.1)	131 (36.6)	0.818
Tricuspid regurgitation				
Yes	11 (33.3%)	1 (11.1%)	12 (28.6%)	0.244
No	3 (9.1%)	2 (22.2%)	5 (11.9%)	
Not assessed	19 (57.6%)	6 (66.7%)	25 (59.5%)	
Late gadolinium enhancement				
Yes	22 (66.7%)	4 (44.4%)	26 (61.9%)	0.329
No	9 (27.3%)	5 (55.6%)	14 (33.3%)	
Not assessed	2 (6.1%)	0 (0%)	2 (4.8%)	

Abbreviations: CMR, cardiac magnetic resonance; LVAD, left ventricular assist devices; LVEF, left ventricular ejection fraction; RVEF, right ventricular ejection fraction; SD, standard deviation; TAPSE, tricuspid annular plane systolic excursion.

Our findings can be summarized as follows: (1) CMR-RVEF did not predict post-LVAD RHF in our cohort, (2) the mean CMR-RVEF was severely reduced in both cohorts with mean CMR-RVEF of 25 ± 13%, (3) the patients who had post-LVAD RHF had larger RV size on CMR; however, this did not achieve statistical significance.

In our cohort, baseline CMR-RVEF prior to LVAD implantation was severely reduced in the patients who developed post-LVAD RHF and those who did not, with no statistically significant difference between the 2 groups. This could be explained by (1) the RV systolic function is dynamic and sensitive to afterload and volume status and, therefore, could change over time, (2) patients are often supported by inotropes prior to LVAD implantation, and this could affect the RV systolic function assessment, with 88% of our cohort were supported by either milrinone or dobutamine prior to LVAD implementation, (3) perioperative factors and thoracotomy approach can influence immediate post-LVAD RHF,^5^ and (4) CMR-derived RV assessment is a snapshot that depicts the RV function at one point of time, the mean time from CMR to LVAD implantation in our cohort was about 7 months. Interestingly, about 80% of the patients in our study had no post-LVAD RHF despite severely reducing CMR-RVEF at the time of CMR.

The study results should be interpreted considering the following limitations. First, there is a selection bias in patients who were able to undergo the CMR exam, including patients who can lay flat and had no significant orthopnea, had less implantable cardioverter-defibrillators, and probably at earlier heart failure stages. Our cohort represents younger patients with a mean age of 47 ± 14.4, compared to the mean age of 57.0 ± 12.8 in the Interagency Registry for Mechanically Assisted Circulatory Support Database.^6^ Compared to prior studies, the incidence of post-LVAD RHF in our analysis was lower at 21%. A meta-analysis revealed that the prevalence of post-LVAD RVF was 35%.^7^ Most of our study's patients (69%) had nonischemic cardiomyopathy. Our cohort represents an overall lower-risk group, limiting the generalizability of the study's results. Second, this is a retrospective study with all the inherent retrospective study biases. Third, the small sample size could underpower the study to assess clinical outcomes; however, this study is intended to be a “proof concept” study.

In conclusion, in patients who underwent CMR prior to LVAD implantation, CMR-derived RVEF may not predict post-LVAD RHF in our cohort. Large multicenter studies are needed to confirm this finding.

## Disclosure statement

The authors declare that they have no known competing financial interests or personal relationships that could have appeared to influence the work reported in this paper.

None of the authors report any funding related to this work. No disclosures to report for any of the authors.
