# Supplementary material for: Role of cardiac MRI in predicting the risk of right heart failure in patients who underwent left ventricular assist device implantation
Source: JHLT Open. 2024 Jan 15;4:100056. doi: 10.1016/j.jhlto.2024.100056 (PMC11935318; doi:10.1016/j.jhlto.2024.100056)
Supplement: Supplementary file 1 — Supplementary material [file mmc1.docx]

**Supplementary Table 1. Baseline demographic data, comorbidities, medications, laboratory data, and clinical outcomes.**

|  | **No right heart failure (N=33)** | **Right heart failure (N=9)** | **Overall (N=42)** | **P-value** |
| --- | --- | --- | --- | --- |
| **Age** |  | | | |
| Mean (SD) | 48.7 (12.6) | 40.8 (19.1) | 47.0 (14.4) | 0.192 |
| **Gender** |  | | | |
| Female | 8 (24.2%) | 2 (22.2%) | 10 (23.8%) | 1 |
| Male | 25 (75.8%) | 7 (77.8%) | 32 (76.2%) |  |
| **Race** |  | | | |
| Asian | 0 (0%) | 0 (0%) | 0 (0%) | 0.371 |
| Native Hawaiian or Other Pacific Islander | 0 (0%) | 0 (0%) | 0 (0%) |  |
| American Indian | 0 (0%) | 1 (11.1%) | 1 (2.4%) |  |
| Black or African American | 10 (30.3%) | 2 (22.2%) | 12 (28.6%) |  |
| White or Caucasian | 20 (60.6%) | 6 (66.7%) | 26 (61.9%) |  |
| Other | 3 (9.1%) | 0 (0%) | 3 (7.1%) |  |
| Unknown/Not Recorded | 0 (0%) | 0 (0%) | 0 (0%) |  |
| **Ethnicity** |  | | | |
| Hispanic or Latino | 3 (9.1%) | 0 (0%) | 3 (7.1%) | 1 |
| Not Hispanic or Latino | 30 (90.9%) | 9 (100%) | 39 (92.9%) |  |
| **BMI** |  | | | |
| Mean (SD) | 30.6 (5.31) | 30.7 (8.99) | 30.6 (6.14) | 0.854 |
| **BSA** |  | | | |
| Mean (SD) | 2.16 (0.224) | 2.09 (0.372) | 2.15 (0.259) | 0.486 |
| **Diabetes** |  | | | |
| Yes | 13 (39.4%) | 3 (33.3%) | 16 (38.1%) | 1 |
| No | 20 (60.6%) | 6 (66.7%) | 26 (61.9%) |  |
| **Hypertension** |  | | | |
| Yes | 12 (36.4%) | 2 (22.2%) | 14 (33.3%) | 0.692 |
| No | 21 (63.6%) | 7 (77.8%) | 28 (66.7%) |  |
| **Chronic Kidney Disease** |  | | | |
| CKD stage I-II | 1 (3.0%) | 1 (11.1%) | 2 (4.8%) | 0.409 |
| CKD stage III-V | 8 (24.2%) | 3 (33.3%) | 11 (26.2%) |  |
| No | 24 (72.7%) | 5 (55.6%) | 29 (69.0%) |  |
| **Cardiomyopathy** |  | | | |
| Ischemic | 11 (33.3%) | 2 (22.2%) | 13 (31.0%) | 0.695 |
| Non-Ischemic | 22 (66.7%) | 7 (77.8%) | 29 (69.0%) |  |
| **Atrial fibrillation** |  | | | |
| Yes | 9 (27.3%) | 2 (22.2%) | 11 (26.2%) | 1 |
| No | 24 (72.7%) | 7 (77.8%) | 31 (73.8%) |  |
| **Ventricular arrhythmia** |  | | | |
| Yes | 10 (30.3%) | 4 (44.4%) | 14 (33.3%) | 0.451 |
| No | 23 (69.7%) | 5 (55.6%) | 28 (66.7%) |  |
| **Smoking** |  | | | |
| Yes | 14 (42.4%) | 6 (66.7%) | 20 (47.6%) | 0.269 |
| No | 19 (57.6%) | 3 (33.3%) | 22 (52.4%) |  |
| **Statin** |  | | | |
| Yes | 17 (51.5%) | 4 (44.4%) | 21 (50.0%) | 1 |
| No | 16 (48.5%) | 5 (55.6%) | 21 (50.0%) |  |
| **Aspirin** |  | | | |
| Yes | 19 (57.6%) | 3 (33.3%) | 22 (52.4%) | 0.269 |
| No | 14 (42.4%) | 6 (66.7%) | 20 (47.6%) |  |
| **Beta Blocker** |  | | | |
| Yes | 16 (48.5%) | 3 (33.3%) | 19 (45.2%) | 0.477 |
| No | 17 (51.5%) | 6 (66.7%) | 23 (54.8%) |  |
| **ACE-I, ARB or Entresto** |  | | | |
| ACE-I | 1 (3.0%) | 2 (22.2%) | 3 (7.1%) | 0.0755 |
| ARB | 6 (18.2%) | 0 (0%) | 6 (14.3%) |  |
| Entresto | 5 (15.2%) | 0 (0%) | 5 (11.9%) |  |
| No | 21 (63.6%) | 7 (77.8%) | 28 (66.7%) |  |
| **Aldosterone antagonist (MRA)/Spironolactone** |  | | | |
| Yes | 28 (84.8%) | 6 (66.7%) | 34 (81.0%) | 0.336 |
| No | 5 (15.2%) | 3 (33.3%) | 8 (19.0%) |  |
| **SGLT-2-I** |  | | | |
| Yes | 0 (0%) | 0 (0%) | 0 (0%) | 1 |
| No | 33 (100%) | 9 (100%) | 42 (100%) |  |
| **Insulin** |  | | | |
| Yes | 3 (9.1%) | 1 (11.1%) | 4 (9.5%) | 1 |
| No | 30 (90.9%) | 8 (88.9%) | 38 (90.5%) |  |
| **Diuretics** |  | | | |
| Yes | 29 (87.9%) | 8 (88.9%) | 37 (88.1%) | 1 |
| No | 4 (12.1%) | 1 (11.1%) | 5 (11.9%) |  |
| **Inotrope** |  | | | |
| Dobutamine | 6 (18.2%) | 2 (22.2%) | 8 (19.0%) | 0.423 |
| Milrinone | 24 (72.7%) | 5 (55.6%) | 29 (69.0%) |  |
| No | 1 (3.0%) | 0 (0%) | 1 (2.4%) |  |
| Vasopressor | 2 (6.1%) | 2 (22.2%) | 4 (9.5%) |  |
| **Anticoagulation** |  | | | |
| DOAC | 10 (30.3%) | 2 (22.2%) | 12 (28.6%) | 0.199 |
| No | 11 (33.3%) | 6 (66.7%) | 17 (40.5%) |  |
| Warfarin | 12 (36.4%) | 1 (11.1%) | 13 (31.0%) |  |
| **Temporary mechanical circulatory support** |  | | | |
| IABP | 2 (6.1%) | 0 (0%) | 2 (4.8%) | 0.634 |
| Impella | 1 (3.0%) | 1 (11.1%) | 2 (4.8%) |  |
| No | 30 (90.9%) | 8 (88.9%) | 38 (90.5%) |  |
| **Sodium** |  | | | |
| Mean (SD) | 136 (2.77) | 135 (2.60) | 136 (2.76) | 0.13 |
| **Creatinine** |  | | | |
| Mean (SD) | 1.16 (0.320) | 1.29 (0.434) | 1.19 (0.346) | 0.451 |
| **BUN** |  | | | |
| Mean (SD) | 20.7 (9.77) | 22.4 (8.63) | 21.1 (9.46) | 0.623 |
| **Albumin** |  | | | |
| Mean (SD) | 3.79 (0.434) | 3.78 (0.492) | 3.79 (0.441) | 0.951 |
| **AST** |  | | | |
| Mean (SD) | 36.2 (31.1) | 119 (203) | 53.8 (100) | 0.89 |
| **ALT** |  | | | |
| Mean (SD) | 48.5 (56.5) | 172 (312) | 75.0 (155) | 0.668 |
| **Alkaline phosphatase** |  | | | |
| Mean (SD) | 87.6 (92.2) | 73.8 (19.0) | 84.7 (82.1) | 0.939 |
| **T Bilirubin** |  | | | |
| Mean (SD) | 0.945 (0.413) | 1.04 (0.856) | 0.967 (0.527) | 0.622 |
| **INR** |  | | | |
| Mean (SD) | 1.33 (0.425) | 1.37 (0.377) | 1.34 (0.411) | 0.839 |
| **Hemoglobin** |  | | | |
| Mean (SD) | 12.5 (1.75) | 12.8 (2.53) | 12.6 (1.91) | 0.53 |
| **Platelets** |  | | | |
| Mean (SD) | 221 (67.0) | 188 (51.7) | 214 (64.9) | 0.163 |
| **Hemoglobin A1c** |  | | | |
| Mean (SD) | 6.16 (1.04) | 5.90 (0.912) | 6.11 (1.01) | 0.667 |
| **BNP** |  | | | |
| Mean (SD) | 1000 (1060) | 920 (571) | 985 (966) | 0.54 |
| **LVAD Type** |  | | | |
| HeartWare | 12 (36.4%) | 6 (66.7%) | 18 (42.9%) | 0.123 |
| HeartMate II | 2 (6.1%) | 1 (11.1%) | 3 (7.1%) |  |
| HeartMate III | 19 (57.6%) | 2 (22.2%) | 21 (50.0%) |  |
| **LVAD strategy** |  | | | |
| BTT | 18 (54.5%) | 2 (22.2%) | 20 (47.6%) | 0.135 |
| DT | 15 (45.5%) | 7 (77.8%) | 22 (52.4%) |  |
| **The total duration of hospital stay after LVAD (days)** |  | | | |
| Mean (SD) | 13.1 (15.9) | 14.4 (6.82) | 13.4 (14.4) | 0.102 |
| **The total duration of intubation after LVAD (days)** |  | | | |
| Mean (SD) | 1.61 (1.12) | 3.00 (3.77) | 1.90 (2.02) | 0.876 |
| **Number of HF rehospitalizations** |  | | | |
| Mean (SD) | 0.727 (1.51) | 2.67 (2.24) | 1.14 (1.84) | 0.00143 |
| **Heart Transplant** |  | | | |
| Yes | 16 (48.5%) | 2 (22.2%) | 18 (42.9%) | 0.258 |
| No | 17 (51.5%) | 7 (77.8%) | 24 (57.1%) |  |
| **Survival status** |  | | | |
| Alive | 28 (84.8%) | 6 (66.7%) | 34 (81.0%) | 0.336 |
| Deceased | 5 (15.2%) | 3 (33.3%) | 8 (19.0%) |  |
