# Supplementary material for: Role of cardiac MRI in predicting the risk of right heart failure in patients who underwent left ventricular assist device implantation
Source: JHLT Open. 2024 Jan 15;4:100056. doi: 10.1016/j.jhlto.2024.100056 (PMC11935318; doi:10.1016/j.jhlto.2024.100056)
Supplement: Supplementary file 2 — Supplementary material [file mmc2.docx]

**Supplementary Table 2. Echocardiography Characteristics**

|  | **No right heart failure (N=33)** | **Right heart failure (N=9)** | **Overall (N=42)** | **P-value** |
| --- | --- | --- | --- | --- |
| **RV systolic function** |  | | | |
| normal | 16 (48.5%) | 1 (11.1%) | 17 (40.5%) | 0.0538 |
| Mild RV systolic dysfunction | 4 (12.1%) | 3 (33.3%) | 7 (16.7%) |  |
| Moderate RV systolic dysfunction | 11 (33.3%) | 3 (33.3%) | 14 (33.3%) |  |
| Severe RV systolic dysfunction | 2 (6.1%) | 1 (11.1%) | 3 (7.1%) |  |
| not well visualized | 0 (0%) | 1 (11.1%) | 1 (2.4%) |  |
| **RV dilation** |  | | | |
| normal | 12 (36.4%) | 2 (22.2%) | 14 (33.3%) | 0.49 |
| Mild RV dilation | 18 (54.5%) | 5 (55.6%) | 23 (54.8%) |  |
| Moderate RV dilation | 2 (6.1%) | 1 (11.1%) | 3 (7.1%) |  |
| Severe RV dilation | 0 (0%) | 0 (0%) | 0 (0%) |  |
| not well visualized | 1 (3.0%) | 1 (11.1%) | 2 (4.8%) |  |
| **Tricuspid regurgitation** |  | | | |
| normal | 2 (6.1%) | 1 (11.1%) | 3 (7.1%) | 0.933 |
| Trace | 12 (36.4%) | 4 (44.4%) | 16 (38.1%) |  |
| Mild | 13 (39.4%) | 3 (33.3%) | 16 (38.1%) |  |
| Moderate | 3 (9.1%) | 0 (0%) | 3 (7.1%) |  |
| Severe | 2 (6.1%) | 1 (11.1%) | 3 (7.1%) |  |
| Not well visualized | 1 (3.0%) | 0 (0%) | 1 (2.4%) |  |
| **LVEF** |  | | | |
| Mean (SD) | 17.5 (7.15) | 13.9 (6.51) | 16.7 (7.10) | 0.0987 |
| **TAPSE (mm)** |  | | | |
| Mean (SD) | 16.6 (4.36) | 14.9 (2.85) | 16.3 (4.14) | 0.285 |
| **Left ventricle end-diastolic dimension (cm) (LVIDd)** |  | | | |
| Mean (SD) | 6.35 (0.694) | 6.54 (0.559) | 6.39 (0.666) | 0.373 |
| **Left ventricular end-diastolic volume index (ml/m2)** |  | | | |
| Mean (SD) | 107 (30.9) | 89.0 (48.1) | 106 (31.5) | 0.611 |
| **RV base diameter (cm)** |  | | | |
| Mean (SD) | 4.39 (0.799) | 4.76 (1.07) | 4.47 (0.865) | 0.355 |
| **PASP: in mmHg** |  | | | |
| Mean (SD) | 42.4 (14.0) | 41.3 (9.18) | 42.2 (13.1) | 0.98 |
