# Supplementary material for: Role of cardiac MRI in predicting the risk of right heart failure in patients who underwent left ventricular assist device implantation
Source: JHLT Open. 2024 Jan 15;4:100056. doi: 10.1016/j.jhlto.2024.100056 (PMC11935318; doi:10.1016/j.jhlto.2024.100056)
Supplement: Supplementary file 3 — Supplementary material [file mmc3.docx]

**Supplementary Table 3. Baseline RHC Characteristics**

|  | **No right heart failure (N=33)** | **Right heart failure (N=9)** | **Overall (N=42)** | **P-value** |
| --- | --- | --- | --- | --- |
| **Systolic blood Pressure (mmHg)** |  | | | |
| Mean (SD) | 112 (18.1) | 101 (18.7) | 109 (18.6) | 0.0726 |
| **Diastolic blood pressure (mmHg)** |  | | | |
| Mean (SD) | 72.8 (10.7) | 65.3 (8.25) | 71.2 (10.6) | 0.0612 |
| **MAP (mmHg)** |  | | | |
| Mean (SD) | 85.4 (11.9) | 76.8 (10.1) | 83.6 (12.0) | 0.0397 |
| **Heart rate (bpm)** |  | | | |
| Mean (SD) | 91.9 (18.9) | 96.6 (14.5) | 92.9 (18.0) | 0.39 |
| **Pulmonary mixed venous saturation (%)** |  | | | |
| Mean (SD) | 59.8 (11.1) | 60.8 (11.0) | 60.0 (10.9) | 0.591 |
| **RA (mmHg)** |  | | | |
| Mean (SD) | 10.9 (7.08) | 9.67 (4.61) | 10.7 (6.60) | 0.842 |
| **RV systolic pressure (mmHg)** |  | | | |
| Mean (SD) | 45.4 (16.5) | 38.9 (8.95) | 44.2 (15.5) | 0.264 |
| **RV end-diastolic pressure (mmHg)** |  | | | |
| Mean (SD) | 10.5 (6.31) | 10.7 (4.61) | 10.5 (5.99) | 0.728 |
| **PA systolic pressure (mmHg)** |  | | | |
| Mean (SD) | 47.5 (16.8) | 41.4 (9.11) | 46.2 (15.6) | 0.342 |
| **PA diastolic pressure (mmHg)** |  | | | |
| Mean (SD) | 24.9 (11.0) | 22.3 (6.73) | 24.3 (10.3) | 0.519 |
| **Mean pulmonary arterial pressure (mmHg)** |  | | | |
| Mean (SD) | 32.1 (12.3) | 28.2 (7.05) | 31.3 (11.4) | 0.325 |
| **PCWP (mmHg)** |  | | | |
| Mean (SD) | 22.4 (9.10) | 21.3 (8.32) | 22.2 (8.85) | 0.728 |
| **TPG (Transpulmonary gradient, mmHg)** |  | | | |
| Mean (SD) | 10.4 (7.25) | 6.89 (2.03) | 9.62 (6.63) | 0.289 |
| **Fick cardiac output (ml/min)** |  | | | |
| Mean (SD) | 4.11 (1.22) | 3.84 (0.947) | 4.05 (1.16) | 0.602 |
| **Thermodilution cardiac output (ml/min)** |  | | | |
| Mean (SD) | 3.77 (1.32) | 3.49 (1.49) | 3.71 (1.34) | 0.517 |
| **Fick cardiac index (ml/min/m2)** |  | | | |
| Mean (SD) | 1.85 (0.514) | 1.83 (0.487) | 1.85 (0.502) | 0.89 |
| **Thermodilution cardiac index (ml/min/m2)** |  | | | |
| Mean (SD) | 1.55 (0.765) | 1.50 (0.860) | 1.54 (0.776) | 0.846 |
| **PVR (Woods Unit)** |  | | | |
| Mean (SD) | 2.62 (1.89) | 1.83 (0.622) | 2.45 (1.73) | 0.416 |
| **SVR (dynes. sec. cm-5)** |  | | | |
| Mean (SD) | 1550 (464) | 1440 (296) | 1530 (433) | 0.83 |
| **PAPi** |  | | | |
| Mean (SD) | 3.82 (4.35) | 3.28 (3.91) | 3.71 (4.22) | 0.434 |
| **RA:PCWP** |  | | | |
| Mean (SD) | 0.457 (0.219) | 0.444 (0.159) | 0.454 (0.206) | 0.838 |
| **RV stroke work index (RVSWI)** |  | | | |
| Mean (SD) | 5.36 (2.80) | 4.67 (2.69) | 5.21 (2.76) | 0.465 |
